# The Benefits of Self-Set Goals: Is Ego Depletion Really a Result of Self-Control Failure?

**DOI:** 10.1371/journal.pone.0157009

**Published:** 2016-06-09

**Authors:** Mario Wenzel, Daniela Zahn, Zarah Rowland, Thomas Kubiak

**Affiliations:** Institute of Psychology, Johannes Gutenberg University, Mainz, Germany; Technion Israel Institute of Technology, ISRAEL

## Abstract

Research on ego depletion aims at explaining self-control failures in daily life. Both resource models and motivational accounts have been proposed for explanation. The aim of the present research was to test the different assumptions in two dual-task experiments where we operationalized ego depletion as a performance deviation from a self-set goal. In two experiments, we found evidence for this deviation contradicting motivational accounts of ego depletion: Participants experiencing ego depletion set themselves a stricter instead of a more lenient goal than controls, in that they chose to eat less cookies or wanted to perform better. Moreover, only participants without an initial self-control task could adhere to their self-set goal, whereas participants in the ego depletion condition in both experiments could not follow through with their more ambitious intentions. Taken together, our findings demonstrate the importance of goals in ego depletion research.

## Introduction

Self-control is usually framed as the ability to control one’s behaviour and its failures are seen as an important factor in individual and societal problems [1]. In the last two decades, the strength model of self-control has been a prominent theory to explain the limits of self-control [2]. According to this model, overriding a dominant response draws on a limited self-regulatory resource. In this state called *ego depletion*, people are not able to spend further self-regulatory resources, resulting in subsequent reduced self-control. One common experimental setup to study ego depletion is a dual-task procedure where controls perform a first task, which does not require self-control and, hence, does not deplete the limited resource. Participants in the experimental condition perform a self-control task as a first task. Since the second task requires self-control in both conditions, a decrease in performance in the experimental condition as compared to controls is due to the increased consumption of the limited resource. There is a plethora of evidence for this ego depletion effect: For example, a meta-analysis by Hagger, Wood, Stiff, and Chatzisarantis [3] reported a medium-to-large pooled effect size of *d* = .62 for the ego depletion effect.

The mechanisms underlying the ego depletion effect observed in subsequent efforts in self-control are widely debated [3]. Ego depletion, for example, does not always occur: Recent research found evidence for moderating factors counteracting ego depletion, such as intrinsic motivation [5] or lay theories about self-control [6]. To acknowledge this evidence, several alternative accounts have been proposed, in which motivation plays a crucial role: Inzlicht and Schmeichel [7] recently presented a reformulated process model where ego depletion results from two interdependent processes: Shifts in motivation and shifts in attention. After initial depletion, people’s motivation shifts away from wanting to control themselves to wanting something rewarding for their efforts, leading to less attention to cues signalling further required self-control. Moreover, de Witt Huberts, Evers, and de Ridder [8] highlighted the role of justifications in self-control failure, where people employ justifications that permit violating goals they endorse to resolve self-control conflict.

Recently, Carter and McCollough [9;10] raised concerns that the pooled effect size of *d* = 0.62 found by Hagger and colleagues [3] may be inflated by small-study effects such as publication bias. Using two correction methods to account for publication bias, they found an average effect size of *d* = 0.25 and *d* = -0.10, respectively. Subsequently, Carter, Kofler, Forster, McCollough [11] conducted a meta-analysis using only laboratory tasks that relate directly to self-control and ego depletion and included non-published effect sizes and found a pooled effect size of *g* = 0.43 that was reduced to zero when accounted for small-study effects. Recently, Hagger and colleagues [12] published the results of a large and rigorous multi-lab replication project of ego depletion that only found a non-significant mean effect size of *d* = 0.04, providing further evidence that the ego depletion effect may not be as robust as reported in the last nearly 20 years.

Thus, since assessing self-control failure by comparing the performance between the experimental and control condition may be limited and the ego depletion may not be reliably replicable, alternative ways of of assessing self-control failures in ego depletion research could help clarify how strongly self-control is limited in laboratory research [13]. A situation requiring self-control is characterized by incompatible motivations, where doing something desired with positive short-term consequences conflicts with a long-term goal [14]. However, ego depletion usually is assessed by a declined performance in the experimental compared to the control condition or to a baseline but only rarely in comparison to a self-set goal. Therefore, the group differences may not reflect a self-control failure, that is a limited capacity to control one’s behaviour, but may also reflect a reduced motivation to control one’s behaviours. To clarify this question, it is necessary to assess the personal goals of the participants with regard to their performance in the second task directly before the second task. In this modified dual-task procedure, ego depletion could not only be operationalized by the performance difference in the second task between experimental and control task but also whether participants in the experimental condition experience more difficulties to adhere to their goal than participants in the control condition. Moreover, this approach enables testing Inzlicht and Schmeichel’s [7] assumption that people shift away from wanting to control themselves after initial depletion which should be reflected in participants setting a more lenient goal in the experimental condition compared to controls. Thus, the aim of the present research was to test the different assumptions made by the strength model of self-control and Inzlicht and Schmeichel [7] in order to disentangle the processes underlying ego depletion. The strength model of self-control would not predict an effect of an initial act of self-control on control-related goals (but possibly on the adherence to those goals), whereas Inzlicht and Schmeichel’s approach [7] would assume decreased control-related goal strength in participants with versus without an initial self-control task.

## Experiment 1

### Method

Both Experiment 1 and 2 were approved by the ethics committee of the Landesärztekammer Rheinland Pfalz (State Chamber of Medicine Rhineland-Palatinate; 837.069.13 (8754-F)). Written informed consent was obtained in both experiments.

#### Participants and design

We recruited 136 students (84 female, age *M* = 24.0 years, *SD* = 3.5) through bulletins and flyers. Each participant received 5 EUR (approx. US$7) as compensation for participating and another 5 EUR as a bonus for completing all experiments. Based on outlier analyses, one participant was excluded due to performance issues in the first task (*z* > 3), six participants due to at least 33% errors in the first task, and one participant due to technical problems. The inclusion of the outliers did not substantially change the results, in that none of the significant results became insignificant and none of the insignificant results were significant without the exclusion of the outliers. Furthermore, a z-test (difference between a coefficient from a model with all participants and a model excluding outliers divided by the square root of the sum of the squared standard errors) did not reveal any significant deviations, *|z|* < 0.22, *p*s > .831. The outlier analyses resulted in a final sample size of 128 students (80 female, age *M* = 23.8 years, *SD* = 3.4). In a between-subject design (ego depletion), participants were randomly assigned to the depletion (*n* = 63) or the control condition (*n* = 65). We aimed for 128 participants to achieve a power of .80 for a t-test for the difference between two independent measures with a medium effect size (*d* = 0.50) and an *α* = .05 and a power of .80 for a within-between interaction in a mixed ANOVA with a small effect size (*d* = 0.25) and an *α* = .05.

#### Procedure

This study was the third of three combined experiments, which were approved by the local ethics committee. The other two experiments were realized one and two weeks before the third experiment and participants were finally debriefed after this experiment. Participants were told not to eat anything for four hours until the experiment started. Prior to the experimental procedure, participants signed an informed consent form and indicated their baseline affect. Then, they completed the first task, which included our ego depletion manipulation. Afterwards, they rated its difficulty and completed the affect questionnaire again. The investigators explained the second task as a product test of cookies and asked the participant how many cookies he or she would like to eat. The investigator made a note of the desired amount, put twice as many cookies on a platter and said that he or she had brought twice as many cookies in case the participant wanted to eat more. Finally, participants had the opportunity to taste the cookies for four minutes and rated the cookies quality and their affect subsequently.

#### Ego depletion manipulation

To manipulate ego depletion, participants in the depletion condition performed the Multi-Source Interference Task (MSIT; [15]), which was developed to maximally stress the conflict-monitoring system ([16]). It was previously used in ego depletion research [17;18]. In the MSIT, participants respond to the character that differs from the other two characters (e.g., “1” in trial “*221*”). The MSIT consists of control (e.g., *xx3*) and interference trials (e.g., *221*, see Bush et al., 2003, for more information). Since, in control trials, the distractors are always the letters x and the place of the target always corresponds to the key participants have to press, there is no response conflict and, thus, control trials do not require self-control. Interference trials comprise several sources of interference, in that the distractors are also numbers and the place of the target does not correspond to the key participants have to press (e.g., *221*). Hence, since only interference trials require self-control, the difference between mean latency on interference trials and control trials serves as a measure of self-control performance.

Participants in the control condition also performed a MSIT but with control trials only. Since responses to control trials are usually faster than to interference trials, the duration of the MSIT in the experimental condition (*M* = 285.5 s, *SD* = 9.4) was longer than in the control condition (*M* = 248.7 s, *SD* = 7.0), *t*(117) = 24.39, *p* < .001, *d* = 4.48. However, including duration in the analyses presented below did not change the results.

In each MSIT trial, a white fixation cross appeared on a black background for 500 ms, followed by the stimulus for a maximum of 2000 ms. The participants responded by pressing predefined keys on a response pad (Cedrus RB730; Cedrus Corporation, San Pedro, CA, USA). Trials hitting the response deadline as well as error trials were excluded from subsequent statistical analyses. The whole experiment consisted of 180 trials, with 36 control and 144 interference trials, presented in the same predetermined random order because we were primarily interested in individual differences.

#### Measures

For the cookie task, participants received twice as many cookies as they wanted and tasted them for four minutes. Afterwards, they rated the cookies’ quality with three items (taste, consistency, and appearance) on a scale from 1 (“very good”) to 6 (“not good at all”). Reliability was acceptable to good, Cronbach’s α = .79. Participants also indicated how often and how willingly they eat cookies in general on a scale ranging from 1 (“not at all”) to 7 (“very”). Reliability was good, with a Spearman-Brown reliability estimate *r* = .74 [19]. Finally, participants responded if they had paid attention not to eat too many cookies and if they had eaten more than they wanted. Our measures of self-control were the amount of cookies eaten by the participants and the deviation of this amount from the amount participants wanted to eat.

To control for group differences in regard to perceived difficulty of the first task, participants rated the three items “difficult”, “frustrating”, and “exhaustive” with a 1 (“not at all”) to 5 (“extremely”) scale. Reliability was marginally acceptable, with a Cronbach’s α of .59.

#### Data analysis

A mixed 2 x 2 ANOVA was conducted with the ego depletion manipulation as between-subject factor and cookies desired vs. cookies eaten as within-subject factor. The interaction between both factors indicates whether our experimental manipulation exhibits any effect on our three interesting variables: cookies eaten, cookies desired and the deviation. If the interaction was significant, simple main effects analysis [20] was performed in order to clarify the effect of the experimental manipulation on those measures.

### Results

#### Preliminary analyses

In order to check whether our manipulation of the first task worked as intended, an independent samples t-test with the depletion measure as the outcome revealed as expected that participants in the depletion condition (*M* = 2.09, *SD* = 0.71) perceived the first task as significantly more difficult than participants in the control condition (*M* = 1.68, *SD* = 0.57), *t*(126) = 3.66, *p* < .001, *d* = 0.65. Moreover, participants in the depletion and control condition did not differ in regard to how they rated the quality of the cookies, *t*(125) = -0.38, *p* = .702, *d* = 0.07, nor how much they enjoy eating cookies in general, *t*(125) = -0.95, *p* = .342, *d* = 0.17. Taken together, these results indicate that our ego depletion manipulation worked as intended and that there were no group differences regarding cookie quality and general preference.

#### Ego depletion and cookies desired

**Fig 1:**
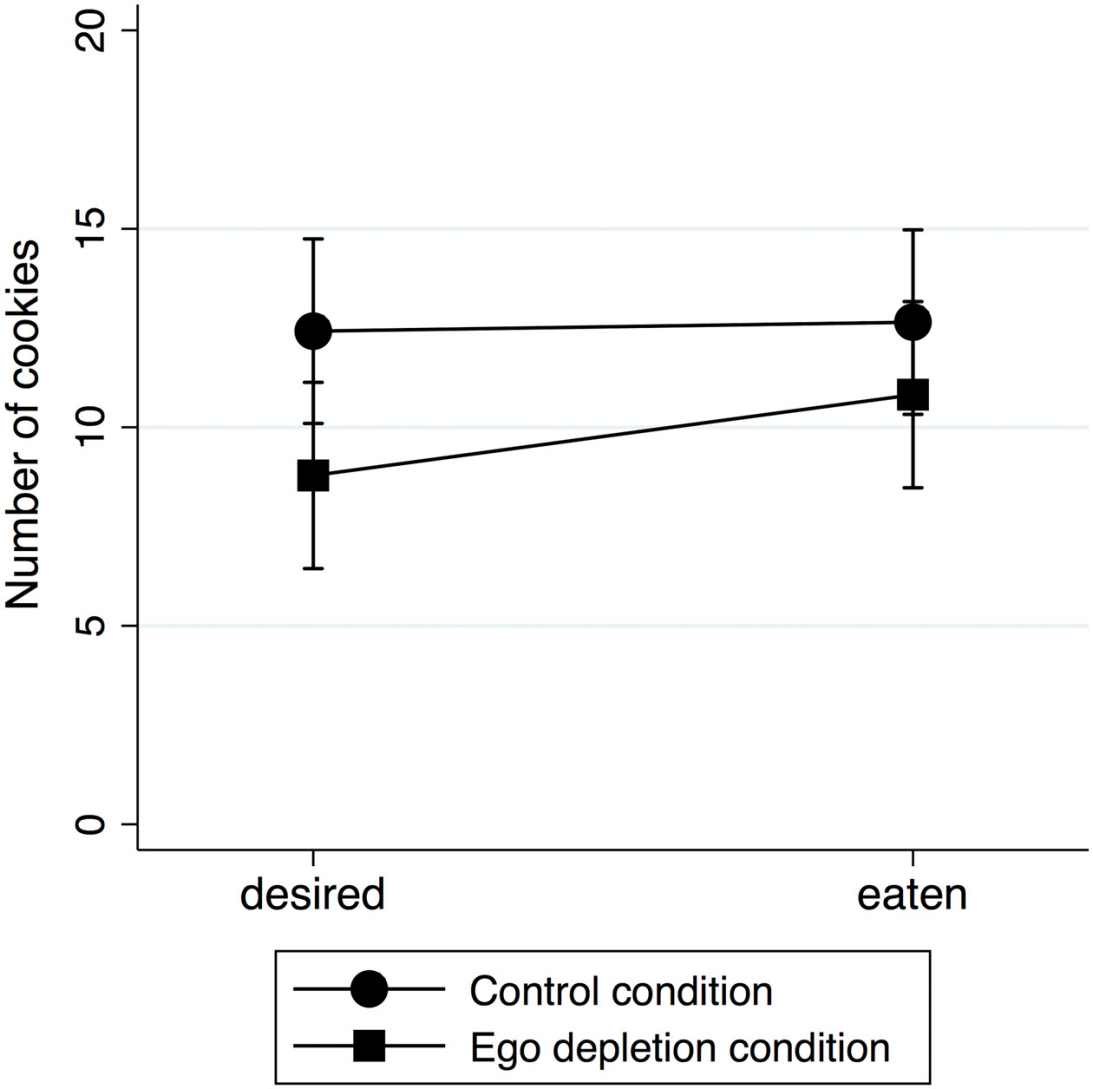
Mean number of cookies desired and eaten as a function of experimental condition (depletion vs. control condition). Error bars represent standard errors.

We computed a 2 (between-subject: ego depletion) x 2 (within-subject: goal deviation: cookies desired vs. cookies eaten) mixed ANOVA. The interaction between ego depletion and deviation from the goal was significant, *F*(1, 126) = 5.52, *p* = .019, η_p_^2^ = .04. This indicates that participants in the depletion and control condition differed in regard to the amount of cookies they wanted to eat and they actually ate and, thus, simple main effects analysis was performed in order to interpret these differences (Fig 1).

First, an independent samples t-test on the number of cookies desired showed that participants in the depletion condition wanted to eat significantly fewer cookies (*M* = 9.33, *SD* = 7.46) than participants in the control condition (*M* = 12.92, *SD* = 12.29), *t*(126) = -1.99, *p* = .049, *d* = -0.35. Thus, the motivation to control one’s eating behaviour was not reduced but increased after initial depletion.

#### Ego depletion and cookies eaten

Furthermore, an independent samples t-test on the number of cookies eaten revealed that participants in the depletion condition (*M* = 11.78, *SD* = 9.71) did not eat more cookies than participants in the control condition (*M* = 13.02, *SD* = 9.77), *t*(126) = 0.72, *p* = .474, *d* = 0.13, indicating no evidence for the typical ego depletion effect.

#### Ego depletion and goal deviation

However, a paired t-test showed on the number of cookies eaten and desired indicated that only participants in the control condition ate the desired amount of cookies, *t*(64) = -0.11, *p* = .911, *d* = -0.02 with dependency-corrected effect size [21], whereas participants in the depletion condition ate significantly more cookies than they wanted, *t*(62) = 4.41, *p* = .001, *d* = 0.67. Importantly, these results did not substantially change when the analyses were controlled for gender and for how often and how willingly the participants eat cookies in general. However, when gender was entered as a factor that could interact with ego depletion and goal deviation, the three-way interaction was significant, *F*(1, 122) = 5.25, *p* = .022, η_p_^2^ = .04. Post-hoc analyses revealed that the two-way interaction was only significant for men, *F*(1, 44) = 5.22, *p* = .022, η_p_^2^ = .11, but not women, *F*(1, 76) = 0.70, *p* = .402, η_p_^2^ = .01.

To summarize, although participants in the depletion condition did not eat more cookies than controls, only participants in the depletion but not in the control condition ate more than they wanted. Moreover, the motivation to control one’s eating behaviour was not reduced after initial depletion, as expected by motivational accounts of ego depletion. By contrast, it was notably increased, in that participants in the experimental condition wanted to eat fewer (not more) cookies than controls. Thus, other than assumed by Inzlicht and Schmeichel [7], participants in the depletion condition set themselves a stricter instead of a more lenient standard than participants in the control condition, indicating stronger intentions to self-control. Next, we conducted Experiment 2 in order to investigate the influence of motivation on self-control performance in more detail.

## Experiment 2

The aim of Experiment 2 was threefold. First, since the results of Experiment 1 were quite surprising, in that we found neither evidence for the strength model of self-control or the assumptions made by Inzlicht and Schmeichel [7] but found that goal strength is higher not lower in the experimental compared to the control condition, we deemed it necessary to conduct a conceptual replication in order to (a) provide further evidence for this effect and (b) generalize the findings of Experiment 1 using an experimental design suitable for many typical self-control tasks, increasing external validity. Since these tasks usually are more artificial than tasks and actions occurring in daily life, such as eating, people may not know how they can or would like to perform in them. Thus, we split the second task in the dual-task procedure in two parts and asked the participants in between how well they want to perform in the second part. Importantly, it might be that splitting the task may induce ego depletion since performing the first task may drain the self-regulatory resource, leading to worse performance in the second part. However, there is evidence that performing a task twice in rapid succession does not lead to ego depletion [22;23].

Second, we wanted to separate goal content from motivation, since individuals may set a goal without the necessary motivation to stick to it. We, thus, included a motivation factor, where participants in the motivation condition received an external reward (chance to win a lottery), to investigate whether a mild induction of external motivation could counteract the deviation from the self-set goal.

Third, we sought to assess motivational and attentional shifts in self-control, as proposed by Inzlicht and Schmeichel [7], more elaborately. To this end, we developed a modified Stroop task designed to capture attention to neutral and motivational distractors. The standard Stroop task comprises two different types of trials: control trials, where the meaning of the word corresponds to the colour the word is presented in, and interference trials, where the meaning of the word does not depict the color. We extended this by two additional types that add a distracting background, which was either neutral (e.g., a table) or motivational (e.g., money) and, thus, captured attention to neutral or rewarding stimuli. If ego depletion is due to shifts in motivation and attention to reward, as proposed by Inzlicht and Schmeichel [7], participants with initial depletion should pay more attention to motivational distractors, leading to increased latency and, thus, larger Stroop effects.

### Method

#### Participants and design

We recruited 100 students (87 female, age *M* = 22.8 years, *SD* = 6.2) through a bulletin. Participants received partial course credit as compensation for participating. Outlier analyses did not reveal substantial outliers. Experiment 2 followed a 2 (ego depletion) x 2 (motivation) between-subject experimental design. We aimed for approximately 100 participants to achieve a power of .80 for an ANOVA with a comparable effect size typically found in ego depletion research of *d* = 0.62 [3] and an α = .05.

#### Procedure

After signing an informed consent form, participants indicated their baseline affect and completed the first task where all participants copied a text by hand but participants in the depletion condition had to substitute each letter e with the number 3, which constituted our ego depletion manipulation. This task resembles a computerized version of the crossing-out-letter task, which demonstrated the largest effect sizes in prior research on ego depletion [3]. In both tasks, participants need to cross out or replace a letter according to certain rules. Since substituting is slower, the text in the control condition was extended to achieve a comparable duration of the first task, which was nearly the case, *t*(98) = 1.72, *p* = .088, *d* = 0.35. Afterwards, participants rated the difficulty of the first task and completed the affect questionnaire again. Then, participants performed the first part of the Stroop task which was followed by the question how well the participants wanted to perform in the second part of the Stroop task. Participants in the external motivation condition were additionally informed that they would take part in a lottery for a $70 voucher if they complete the second part of the Stroop task as good as the first part. Finally, participants rated their affect again.

#### Measures

Stroop stimuli were shown in a green or red font on a black background. Each trial began with a white fixation cross for 800 ms, followed by the stimulus for a maximum of 2000 ms. The participants responded by pressing predefined keys on the keyboard (g for green words and h for red words). All error trials including trials hitting the response deadline were excluded from subsequent statistical analyses. The first and second part of the Stroop task consisted of 70 trials each; with 18 control trials, 18 interference trials, 17 neutral distraction interference trials, and 17 motivational distraction interference trials in a predetermined random order since we were primarily interested in individual differences.

Goal strength for performing well in the second part of the Stroop task was assessed by the single item “How well would you like to perform in the second part of task compared to the first part?” on a 7-point scale ranging from -3 (“worse”) to 3 (“better”). On average, participants wanted to perform better than in the first part (*M* = 1.14, *SD* = 1.13), with three participants indicating a negative goal strength and 71 participants a positive goal strength.

To achieve a better check for our depletion manipulation, we kept the item “exhaustive” and replaced the items “difficult” and “frustrating” with “taxing”, assessed on a 1 (“not at all”) to 7 (“extremely”) scale. Reliability of the average score was good, with a Spearman-Brown reliability estimate of .79 for the first task and .81 for the second [19].

### Results

#### Preliminary analyses

Participants in the depletion condition perceived the first task as more exhaustive and taxing (*M* = 4.12, *SD* = 1.08) than participants in the control condition (*M* = 3.31, *SD* = 1.43), as evidenced by a significant independent samples t-test on the depletion manipulation check, *t*(98) = 3.18, *p* = .002, *d* = 0.64. For the second task, this effect did not reach significance, *t*(98) = 1.56, *p* = .121, *d* = 0.32. This indicates that our ego depletion manipulation worked as intended.

#### Ego depletion and goal strength

As in Experiment 1, we first investigated whether our ego depletion manipulation exhibited any effects on Stroop performance, goal strength and the goal deviation. Afterwards, we report the effects of the motivation manipulation on those outcomes.

First, we investigated whether participants differed in the goal they set themselves regarding how well they wanted to perform in the second part of the Stroop task. To this end, we computed a 2 (ego depletion) x 2 (motivation) ANOVA on goal strength, which yielded a significant main effect of ego depletion, *F*(1, 96) = 4.84, *p* = .030, η_p_^2^ = .05. Participants in the depletion condition reported significantly stronger goal intentions (*M* = 1.41, *SD* = 0.98) than controls (*M* = 0.92, *SD* = 1.21), which mirrors the results of Experiment 1.

#### Ego depletion and Stroop performance

Next, in order to test for ego depletion effects, we conducted a 2 (ego depletion) x 2 (motivation) ANOVA on Stroop performance in the second task. As illustrated in Table 1, which shows the simple main effect using an independent samples t-test of the ego depletion factor on three self-control measures of the Stroop task, participants in the depletion condition did not significantly differ in any of the performance measures in both parts the Stroop task compared to controls. As in Experiment 1, we did not found evidence for the typical ego depletion effect, reflected in a difference between experimental and control group.

**Table 1:** Means and standard deviations of the central variables as a function of ego depletion condition.

	Experimental Condition		Control Condition				
	*M*	*SD*	*M*	*SD*	*t*(98)	*p*	*d*
Stroop effect, part 1	18.41	56.79	11.82	46.59	0.64	.526	0.13
Stroop effect, part 2	34.39	31.64	28.80	35.13	0.84	.406	0.17
Incongruent trials, part 1	483.87	72.67	484.91	94.30	-0.06	.951	-0.01
Incongruent trials, part 2	453.45	61.40	442.65	57.66	0.91	.367	0.18
Errors, part 1	3.98	3.21	3.75	3.90	0.33	.744	0.07
Errors, part 2	2.41	2.01	2.10	2.11	0.75	.454	0.15

*N* = 100. p-values are two-tailed.

#### Ego depletion and goal deviation

In order to investigate goal deviation, we conducted a 2 (ego depletion) x 2 (motivation) ANCOVA on the latency of incongruent trials in the second part of the Stroop task and included goal strength (centered) and its interactions as continuous variables and differences in the latency of incongruent trials in the first part as a control variable. We focused on latency of incongruent trials only since the quicker adaptation to congruent trials in the second part of the Stroop task hampers the interpretation of the Stroop effect. As indicated in Table 2, goal strength interacted significantly with ego depletion, *p* = .008. Simple slope analysis [20] revealed that participants in the depletion condition responded significantly slower to incongruent trials, *b* = 14.51, *SE* = 6.51, *t*(91) = 2.23, *p* = .028, indicating that they were significantly less able to follow through with their intentions. In contrast, performance of participants in the control condition was not influenced by goal strength, *b* = -8.41, *SE* = 5.29, *t*(91) = -1.59, *p* = .116. Thus, although participants in the depletion condition wanted to perform better than before in the second part of the Stroop task compared to controls, they could not keep up with their more ambitious goal.

**Table 2:** Analysis of covariance (ANCOVA) between ego depletion condition, manipulation condition and goal strength.

	*F*	*df*	*p*	η_p_^2^
Ego depletion	1.80	1	.184	.02
Motivation	0.02	1	.894	.00
Goal strength	0.46	1	.501	.01
Ego depletion x motivation	0.41	1	.523	.01
Ego depletion x goal strength	7.36	1	.008	.08
Motivation x goal strength	7.31	1	.008	.07
Ego depletion x motivation x goal strength	1.84	1	.178	.02
Latency incongruent trials, part 1	73.08	1	< .001	.45

*N* = 100. *p*-values are two-tailed.

#### Ego depletion and motivation

As illustrated in Table 2, the motivation condition and the interaction with ego depletion did not exhibit a significant effect on Stroop performance in the 2 x 2 (ego depletion) x 2 (motivation) ANCOVA. However, like the ego depletion condition, the motivation condition significantly interacted with goal strength, in that participants with an external reward could keep up with their more ambitious goals, *b* = -8.63, *SE* = 5.45, *t*(91) = -1.58, *p* = .117, whereas controls could not, *b* = 13.83, *SE* = 6.32, *t*(91) = 2.19, *p* = .031. Although the three-way interaction between ego depletion, motivation, and goal strength was not significant, *F*(1, 91) = 1.84, *p* = .178, η_p_^2^ = .02, simple slope analysis revealed the predicted pattern: When participants had the chance to take part in the lottery, both participants in the depletion and control condition performed better the higher they intentions were, although only the simple slope for participants in the control condition reached significance, *b* = -2.85, *SE* = 8.37, *t*(91) = -0.34, *p* = .734 and *b* = -14.19, *SE* = 6.42, *t*(91) = -2.21, *p* = .030. Without external motivation, performance of participants in the control condition was not significantly influenced by goal strength, *b* = -2.85, *SE* = 8.88, *t*(91) = -0.32, *p* = .749, but participants in the experimental condition showed difficulties keeping up with their intention, *b* = 31.19, *SE* = 9.48, *t*(91) = 3.29, *p* = .001. The same analysis with the number of errors showed a similar pattern: Both ego depletion, *F*(1, 91) = 8.07, *p* = .005, η_p_^2^ = .08, and motivation, *F*(1, 91) = 4.08, *p* = .046, η_p_^2^ = .04, interacted with goal strength significantly. The results did not substantially change when the analyses were controlled for gender. Thus, only participants in the depletion condition demonstrated difficulties in keeping up with their intentions as in Experiment 1, especially without external reward.

#### Attentional and motivational processes

**Fig 2:**
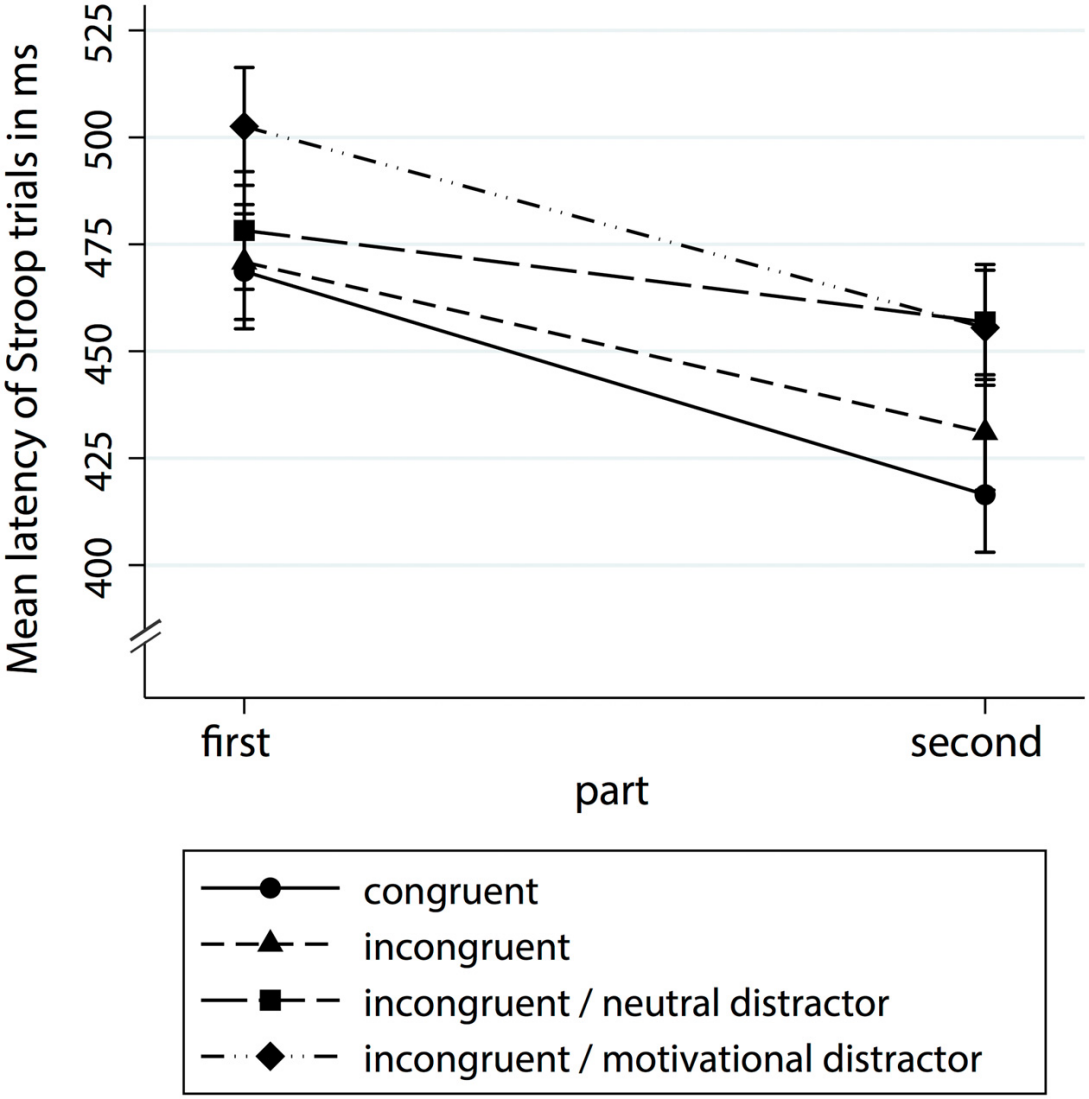
Mean latency of Stroop trials as a function of Stroop task part and trial type. Error bars represent standard errors.

Inzlicht and Schmeichel [7] hypothesized that decreased performance after initial self-control may be due to increased attention to rewarding stimuli, which interferes with task demands. To investigate those attentional shifts in our modified Stroop task, we computed a multilevel model with latency as the outcome and simultaneously entering depletion and motivation conditions, trial type (congruent vs. incongruent vs. incongruent with neutral distractor vs. incongruent with motivational distractor), and part of the Stroop task as the predictors using the mixed command in Stata 13 (Stata Corporation, College Station, TX, USA). This model revealed a main effect for trial type, *χ*^2^(1, *N* = 13,335) = 150.24, *p* < .001. Responses to congruent trials were the fastest (*M* = 441.45 ms, *SE* = 6.49), followed by incongruent trials (450.08 ms, *SE* = 6.49), incongruent trials with neutral distractor (*M* = 467.08 ms, *SE* = 6.53), and incongruent trials with motivational distractor (*M* = 478.02 ms, *SE* = 6.53). All Šidák-adjusted group comparisons were significant. Moreover, the part of the Stroop task also was significant, *χ*^2^(1, *N* = 13,335) = 302.48, *p* < .001, with participants responding faster in general in the second part. Importantly, the part of the Stroop task also interacted with the trial type, *χ*^2^(3, *N* = 13,335) = 26.06, *p* < .001. Analysis of marginal means (see Fig 2) indicated that whereas in the first part of the Stroop task, congruent trials, incongruent trials and incongruent trials with neutral distractor did not differ significantly from each other, *t*s(13,203) < 1.99, *p*s > .747 (Šidák-adjusted), mean latency on incongruent trials with motivational distractors was significantly higher compared to the other trial types, most importantly incongruent trials with neutral distractors, *t*(13,203) = 4.98, *p* < .001. In the second part of the Stroop task, this difference disappeared, *t*(13,203) = -0.31, *p* = 1.00. However, none of the experimental conditions influenced this two-way interaction significantly, *χ*^2^s(1, *N* = 13,335) < 3.63, *p*s < .304, which demonstrates that not only participants with initial depletion paid more attention to rewarding stimuli.

## General Discussion

We investigated the roles of motivation and goal strength in ego depletion in two experiments. We did not find evidence for ego depletion, in that participants with a first requiring self-control in Experiment 1 ate as many cookies and performed equally well in the Stroop task in Experiment 2 than participants with a first task that did not require self-control. However, participants in the depletion condition in Experiment 1 and 2 reported stronger intentions to control themselves than controls. Importantly, participants in the depletion condition in Experiment 1 could not keep up with their goal and ate more than they wanted, whereas participants in the control condition did not deviate significantly from their self-set goal. This effect was conceptually replicated in Experiment 2, in that only participants in the experimental condition and without an additional external reward performed the worse the stricter their goal was.

Thus, we did not find strong support for the process model by Inzlicht and Schmeichel [7]. Whereas other research shows that initial depletion increases approach motivations [24], participants in the depletion condition did not want to eat more, but significantly less cookies (Experiment 1) and wanted to perform better than controls (Experiment 2). In contrast to Schmeichel and colleagues [24], asking people how much they would like to eat taps into both, approach motivation and avoidance motivation (controlling an unwanted behaviour), which could partially explain the different results. However, in Experiment 2, we also found that both groups demonstrated an increased approach motivation at the beginning of the second task, which diminished over the course of the task. Hence, attention to motivational stimuli may not be a proxy for goal strength and the notion of a specific increase after initial depletion could not be confirmed.

Our results are in line with predictions made by the counteractive self-control theory [25]. According to this theory, temptations do not always lure people into failure but may also increase the strength of the goal to control one’s own behaviour in this situation. Thus, initial depletion may have triggered self-control goals that are still activated before and during the second task, resulting in stronger intentions to control the behaviour. In turn, the activated self-control goal led to exerting greater control in the second task, which may have counteracted the typical ego depletion effect. However, participants in the depletion condition were less able to follow through with their more ambitious intentions when faced with increased temptation. Thus, it remains unclear whether ego depletion may involve some sort of self-control failure or whether participants in the depletion condition may set themselves higher standards that are a bit too optimistic. To separate these two explanations, future research should combine research in ego depletion with counteractive self-control theory: For instance, goals could be assessed *before* both tasks in order to examine whether performance in the second task deviates from the baseline goal strength after an initial self-control task, suggesting some sort of ego depletion effect, or whether their goal striving is too optimistic, indicated by a performance in the second task that is in line with the baseline goal but not with an increased goal strength after the first task.

A potential limitation of our study was that we did not find ego depletion effects in both experiments. The absence of a significant ego depletion effect may indicate that our experimental manipulation failed, which could hamper the interpretability of our analyses. However, we not only found similar effect sizes in both experiments compared to Carter and colleagues [11] but we could also show that there are other processes and performance limitation in the dual-task design except for a group difference in the second task. That is, participants in the depletion condition set themselves stricter goals than participants in the control condition, which is not in line with the assumption that initial acts of self-control lead to decreased motivation to control oneself. This effect remained of similar size when participants’ habitual preference for cookies was controlled for in Experiment 1. Moreover, we could find evidence for the predicted shifts in attention towards reward stimuli in Experiment 2; these shifts, however, were independent from our ego depletion manipulation: Even participants in the control condition showed larger Stroop latencies in trials with motivational distractor compared to neutral ones. Finally, our effect sizes in Experiment 1 (*d* = -0.08) and 2 (around *d* = 0.20) are close to the bias-corrected pooled effect size of *d* = 0.25 and *d* = -0.10 reported by Carter and McCollough [9;10]. Thus, our research indicates that assessing the deviation from self-set goals presents an interesting alternative way of assessing self-control failure.

Furthermore, we chose a cookie tasting task in Experiment 1 since consuming sugary food is often tempting and controlled by individuals and individuals can easily report how much they want to eat of it. Although the participants reported that they had paid attention not to eat too many cookies, future research could limit the sample to individuals that restrict their eating behaviour or could use a task where individuals are asked to eat radishes while avoiding cookies in order to increase the self-control conflict.

Another limitation refers to our manipulation check of ego depletion. In the first experiment, we assessed task difficulty, although commonly used in ego depletion research [3], instead of depletion, which we changed in the second experiment to items that better reflect depletion. However, given the importance of assessing initial depletion, developing a scale that can validly differentiate between difficult and depleting tasks would be of utmost importance to advance self-control research.

Although we did not find the typical ego depletion effects, our results suggest that initial acts of self-control involve deviation from a self-set goal. Whether this deviation can be seen as a self-control failure is still unclear. Clearly, more research is needed that compares the assumptions of the resource and motivational approaches, but we believe that assessing ego depletion as a deviation from a self-set goal is a promising method in this endeavour.

## Supporting Information

S1 Dataset Data collected for Experiment 1. (CSV)

S2 Dataset Data collected for Experiment 2. (CSV)

## References

[1] Muraven M, Tice DM, Baumeister RF. Self-control as a limited resource: Regulatory depletion patterns. J Pers Soc Psychol. 1998;74: 774–789. 10.1037/0022-3514.74.3.774 9523419

[2] Baumeister RF. Ego depletion and self-control failure: An energy model of the self’s executive function. Self Identity. 2002;1: 129–136. 10.1080/152988602317319302

[3] Hagger MS, Wood C, Stiff C, Chatzisarantis NLD. Ego depletion and the strength model of self-control: A meta-analysis. Psychol Bull. 2010;136: 496–525. 10.1037/a0019486 20565167

[4] Kurzban R, Duckworth A, Kable J, Myers J. An opportunity cost model of subjective effort and task performance. Behav Brain Sci. 2013;36: 661–726. 10.1017/S0140525X12003196 24304775 PMC3856320

[5] Moller AC, Deci EL, Ryan RM. Choice and ego-depletion: The moderating role of autonomy. Pers Soc Psychol Bull. 2006;32: 1024–1036. 10.1177/0146167206288008 16861307

[6] Job V, Dweck CS, Walton GM. Ego depletion: Is it all in your head? Implicit theories about willpower affect self-regulation. Psychol Sci. 2010;21: 1686–1693. 10.1177/0956797610384745 20876879

[7] Inzlicht M, Schmeichel BJ. What is ego depletion? Toward a mechanistic revision of the resource model of self-control. Perspect Psychol Sci. 2012;7: 450–463. 10.1177/1745691612454134 26168503

[8] De Witt Huberts JC, Evers C, De Ridder DTD. “Because I am worth it”: A theoretical framework and empirical review of a justification-based account of self-regulation failure. Pers Soc Psychol Rev. 2014;18: 119–138 10.1177/1088868313507533 24214148

[9] Carter EC, McCullough ME. Publication bias and the limited strength model of self-control: has the evidence for ego depletion been overestimated? Behav Brain Sci. 2013;36: 683–684. 25126083 10.3389/fpsyg.2014.00823 PMC4115664

[10] Carter EC, McCullough ME. Publication bias and the limited strength model of self-control: has the evidence for ego depletion been overestimated? Front Psychol. 2014;5:823 10.3389/fpsyg.2014.00823 25126083 PMC4115664

[11] Carter EC, Kofler LM, Forster DE, McCullough ME. A series of meta-analytic tests of the depletion effect: Self-control does not seem to rely on a limited resource. J Exp Psychol Gen. 2015;144: 796–815. 10.1037/xge0000083 26076043

[12] Hagger M. S., Chatzisarantis N. L. D., Alberts H., Anggono C. O., Birt A., Brand R., … Rentzsch K., … Zweinenberg M. (in press). A multi-lab pre-registered replication of the ego-depletion effect. *Perspectives on Psychological SciencePerspect Psychol Sci* 2016; in press. 10.1177/1745691616652873 27474142

[13] Converse PD, DeShon RP. A tale of two tasks: Reversing the self-regulatory resource depletion effect. Journal of Applied Psychology. 2009;94: 1318–1324. 10.1037/a0014604 19702373

[14] Hofmann W, Friese M, Strack F. Impulse and self-control from a dual-systems perspective. Perspect Psychol Sci. 2009;4: 162–176. 10.1111/j.1745-6924.2009.01116.x 26158943

[15] Bush G, Shin LM, Holmes J, Rosen BR, Vogt BA. The Multi-Source Interference Task: Validation study with fMRI in individual subjects. Mol Psychiatry. 2003;8: 60–70. 10.1038/sj.mp.4001217 12556909

[16] Botvinick MM, Braver TS, Barch DM, Carter CS, Cohen JD. Conflict monitoring and cognitive control. Psychological Review. 2001; 108: 624–652. 10.1037/0033-295X.108.3.624 11488380

[17] Shamosh N, Gray J. The relation between fluid intelligence and self-regulatory depletion. Cogn Emot. 2007;21: 1833–1843. 10.1080/02699930701273658

[18] Wenzel M, Kubiak T, Conner TS. Positive affect and self-control: Attention to self-control demands mediates the influence of positive affect on consecutive self-control. Cogn Emot. 2014;28: 747–755. 10.1080/02699931.2013.851069 24199679

[19] Eisinga R, Te Grotenhuis M, Pelzer B. The reliability of a two-item scale: Pearson, Cronbach or Spearman-Brown?. International Journal of Public Health. 2012;58: 637–642. 10.1007/s00038-012-0416-3 23089674

[20] Aiken LS, West SG. Multiple regression: Testing and interpreting interactions. 1st ed Thousand Oaks: Sage Publications; 1991.

[21] Morris SB, DeShon RP. Combining effect size estimates in meta-analysis with repeated measures and independent-group designs. Psychol Methods. 2002;7: 105–125. 10.1037/1082-989X.7.1.105 11928886

[22] Dewitte S, Bruyneel S, Geyskens K. Self-regulating enhances self-control in subsequent consumer decisions involving similar response conflicts. J Consum Res. 2009;36: 394–405. 10.1086/598615

[23] Wenzel M, Conner TS, Kubiak T. Understanding the limits of self-control: Positive affect moderates the impact of task switching on consecutive self-control performance. Eur J Soc Psychol. 2013;43: 175–184. 10.1002/ejsp.1936

[24] Schmeichel BJ, Harmon-Jones C, Harmon-Jones E. Exercising self-control increases approach motivation. J Pers Soc Psychol. 2010;99: 162–173. 10.1037/a0019797 20565193

[25] Fishbach A, Zhang Y, Trope Y. Counteractive evaluation: Asymmetric shifts in the implicit value of conflicting motivations. J Exp Soc Psychol. 2010;46: 29–38.

